# Preclinical immunogenicity risk assessment of biotherapeutics using CD4 T cell assays

**DOI:** 10.3389/fimmu.2024.1406040

**Published:** 2024-05-28

**Authors:** Robin E. Walsh, Angela Nix, Chloé Ackaert, Aurélie Mazy, Jana Schockaert, Sofie Pattyn, Laurent Malherbe

**Affiliations:** ^1^ Lilly Research Laboratories, Eli Lilly and Company, Indianapolis, IN, United States; ^2^ ImmunXperts SA| Rue August Piccard 48, Gosselies, Belgium

**Keywords:** immunogenicity, CD4 T cell proliferation, dendritic cells, major histocompatibility complex class II, T cell epitopes, therapeutic proteins

## Abstract

T-cell dependent antibody responses to biotherapeutics remain a challenge to the optimal clinical application of biotherapeutics because of their capacity to impair drug efficacy and their potential to cause safety issues. To minimize this clinical immunogenicity risk, preclinical assays measuring the capacity of biotherapeutics to elicit CD4 T cell response *in vitro* are commonly used. However, there is considerable variability in assay formats and a general poor understanding of their respective predictive value. In this study, we evaluated the performance of three different CD4 T cell proliferation assays in their capacity to predict clinical immunogenicity: a CD8 T cell depleted peripheral blood mononuclear cells (PBMC) assay and two co-culture-based assays between dendritic cells (DCs) and autologous CD4 T cells with or without restimulation with monocytes. A panel of 10 antibodies with a wide range of clinical immunogenicity was selected. The CD8 T cell depleted PBMC assay predicted the clinical immunogenicity in four of the eight highly immunogenic antibodies included in the panel. Similarly, five antibodies with high clinical immunogenicity triggered a response in the DC: CD4 T cell assay but the responses were of lower magnitude than the ones observed in the PBMC assay. Remarkably, three antibodies with high clinical immunogenicity did not trigger any response in either platform. The addition of a monocyte restimulation step to the DC: CD4 T cell assay did not further improve its predictive value. Overall, these results indicate that there are no CD4 T cell assay formats that can predict the clinical immunogenicity of all biotherapeutics and reinforce the need to combine results from various preclinical assays assessing antigen uptake and presentation to fully mitigate the immunogenicity risk of biotherapeutics.

## 1 Introduction

The immunogenicity of biotherapeutics, i.e., their propensity to evoke an unwanted immune response in patients, is an important consideration in drug development because of its potential to influence the safety, efficacy, and overall therapeutic outcomes. Immunogenicity can manifest in various forms, ranging from the production of neutralizing antibodies to hypersensitivity reactions, which can profoundly impact patient health and treatment success. Consequently, understanding, assessing, and mitigating immunogenicity risks through proper design, characterization, and monitoring strategies are essential steps in the development and regulatory approval process of biotherapeutics.

Pharmaceutical companies, biotechnology companies, and contract research organizations are using a variety of approaches to predict clinical immunogenicity. The most common assays are measuring the capacity of biotherapeutics to elicit CD4 T cell responses *in vitro*. CD4 T cells are essential to the development of the anti-drug antibodies (ADA) responses and unlike B cells their responses can readily be assessed *in vitro* (1). While there is general recognition of the importance of CD4 T cells in the ADA response, there is no agreement on assay format to measure CD4 T cell responses (2, 3) Some companies used peripheral blood mononuclear cells (PBMCs) depleted or not of CD8 T cells (4) and/or regulatory T cells (5) while others used a co-culture with monocyte-derived dendritic cells (DCs) (6) and purified CD4 T cells (7). The duration of the T cell assays varies between laboratories ranging from 2 days (8) to 3 weeks (9) while the number of donors evaluated fluctuates between 10 to 50 donors. Finally, diverse endpoints are used to measure CD4 T cell responses including the expression of T cell activation markers (8), T cell proliferation, or the production of cytokines (IL-2, IFN-γ or IL-5). Whether all these CD4 T cell assays predict equally well clinical immunogenicity is however unclear.

In this study, we reviewed the performance of one assay format, the PBMC assay with CD8 T cell depletion, using 45 homologs of clinically tested monoclonal antibodies (mAbs). We then selected 10 mAbs that were correctly predicted or not by the PBMC assay to determine whether DC-based CD4 T cell assays with or without a restimulation step would have a better predictive value.

## 2 Materials and methods

### 2.1 Monoclonal antibodies and proteins

For CD8+ Depleted PBMC assay, the positive assay control (10), keyhole limpet hemocyanin (Imject™ mcKLH), was purchased from Thermo Fisher Scientific and was reconstituted with 2mL of ultrapure water. The final assay concentration for KLH was 0.33µM. KLH used in DC-T assays with (50ug/ml) and without (25ug/ml) stimulation was purchased from Enzo. Proprietary antibodies, mAb1, mAb2, mAb3, mAb4, and mAb5 were supplied by Eli Lilly and Co. Anti-PCSK9-A, anti-PCSK9-B, anti-IL21R, anti-IL7R, and anti-PDL1 homologs were synthesized using the published sequences described by W.H.O. International Nonproprietary Names for Pharmaceutical Substances or U.S. patents. Whole antibody heavy and light chains were subcloned from the VH and VL genes, respectively. The mAbs of interest were produced by transfection into Chinese Hamster Ovary (CHO-GS/Lipase KO(2F9) cells (Lonza, Basel, Switzerland)) cells. A Protein-A affinity chromatography (MabSelect SuRe; GE Healthcare Biosciences, AB, Uppsala, Sweden) and Strong Cation Exchange (SCX/SEC) chromatography (Poros50 HS SCX (Thermo Scientific Cat#1335906, GE Healthcare cat#28922937) were used to purify the respective cell culture fluid for each antibody. The final concentration of all the mAbs used in the assay was 50 µg/ml (0.33 µM).

### 2.2 CD8 T cell depleted PBMC proliferation assay

Cryopreserved PBMCs were purchased from an HLA-DR1 characterized library available through Cellular Technology Limited (CTL; cat# CTL-CP1) and were thawed according to CTL’s instructions using Anti-Aggregate Wash™ Medium (CTL-AA-005). CD8+ T cells were depleted from the PBMCs by immunomagnetic sorting using CD8 Microbeads, human (Miltenyi Biotec, cat # 130–045-201) using an autoMACS Pro separator (Miltenyi Biotec) according to the manufacturer’s protocol. CD8 depleted PBMCs were washed, labeled with 1 µM Carboxyfluorescein Diacetate Succinimidyl Ester (CFSE, Molecular Probes, cat # C34554)), and resuspended in AIM-V media (Life Technologies, cat# 12055–083) containing 5% CTS™ Immune Cell SR (Gibco, cat# A2596101). Using several different 10 donor cohorts, the cells then were seeded at 4 x 10^6^ cells/ml/well and tested in triplicate in 2.0 mL containing the different test articles, KLH, or media control only. After cultures were incubated for 7 days at 37°C with 5% CO_2_, samples were stained with cell surface markers: anti-CD3 (BioLegend, cat# 300424), anti-CD4 (BioLegend, cat# 300530), anti-CD14 (BD Biosciences, cat#563743), anti-CD19 (BD Biosciences, cat#562440), and DAPI (BD Pharmingen, cat#564907) for viability detection by flow cytometry using a BD LSRFortessa™, equipped with a High Throughput Sampler (HTS). FlowJo™ v10.8 Software (BD Life Sciences) was used to analyze data and a Cellular Division Index (CDI) was calculated as described previously (11).

### 2.3 DC: CD4 T cell proliferation assay

HLA-typed PBMCs isolated from 50 healthy donor whole blood according to the ethical protocol/amendment IXP-001_V3 (Belgium; Reg. Nr. B6702014215858), protocol IXP-003_V1 (Belgium; Reg. Nr. B707201627607) or protocol IXP-004_V1 (The Netherlands; Reg. Nr. NL57912.075.16) and were kept in cryogenic storage (-180°C) until use. PBMCs were thawed in culture medium. Monocytes were isolated by magnetic separation (Miltenyi (cat#130–050-201)) and cultured for 5 days in DC medium including interleukin 4 (IL-4, Miltenyi Biotech cat# 130–093-922) and granulocyte-macrophage colony-stimulating factor (GM-CSF, Miltenyi Biotech cat# 130–093-866). On day 5, the monocytes were differentiated into immature DCs (iDCs). The iDCs were collected, seeded into cell culture plates, and then pulsed with mAbs, buffer, or controls, while further cultured in medium supplemented with Interleukin-1 beta (IL-1β) and tumor necrosis factor alpha (TNF-α) for overnight maturation. On day 6, monocyte-derived DCs were washed. Autologous CD4 T cells from the respective donors were isolated by negative magnetic separation according to Manufacturer’s instructions (StemCell: EasySep™ Human CD4+ T Cell Enrichment Kit; 19052) and co-cultured with the antigen loaded DCs for 6 days. To confirm the differentiation process of monocytes into DCs, samples of monocyte cultures were taken on days 0, 5 and 6, respectively. The cells then were fluorescently stained for a set of differentiation and maturation markers (CD14, CD80, CD83, CD86, CD40, CD209 and HLA-DR). CD4 T cell proliferation was assessed by measuring 5-Ethynyl-2´-deoxyuridine incorporation (12). On day 12, the DC-T cell co-culture was pulsed with EdU for approximately 16 hours. Afterwards, the cells were fluorescently stained for live/dead differentiation, T cell surface markers (CD3 and CD4), fixed, permeabilized, and the incorporated EdU was stained with a fluorescent azide. Flow cytometry data were acquired with a BD FACSymphony™ (BD Biosciences) and analyzed using Flowlogic™ software (Innovai, Australia).

### 2.4 DC: CD4 T cell re-stimulation assay

PBMCs isolated from 10 healthy donors were retrieved from cryogenic storage and thawed in culture medium. Monocytes were isolated and differentiated into DCs as previously described. DCs were then seeded onto cell culture plates and then pulsed with the therapeutic mAbs (50 μg/ml), buffer, or controls, while further cultured in medium supplemented with IL-1β and TNF-α for overnight maturation. Autologous CD4 T cells were isolated and co-cultured with the antigen loaded DCs. After 5 days of co-culture the CD4 T cells are harvested and seeded on a FluoroSpot plate with and without restimulation with fresh monocytes and the therapeutic mAbs overnight. Then the next day the plate is developed with IFN-γ and IL-5 antibodies. The stimulation index (SI) was calculated by dividing the average number of spots/1 x 10^6^ cells by the average number of spots observed in the medium control wells. When control wells were negative for Spot Forming Units (SFU), we set the number of negative wells to 1, since the formula cannot accept the value 0 (13, 14).

### 2.5 Statistical analysis

All analysis were performed using GraphPad Prism (version 10) or Excel. The specific statistical tests used are indicated in the figure legends.

## 3 Results

### 3.1 *In vitro* T cell assays used in preclinical immunogenicity risk assessment

**Figure 1:**
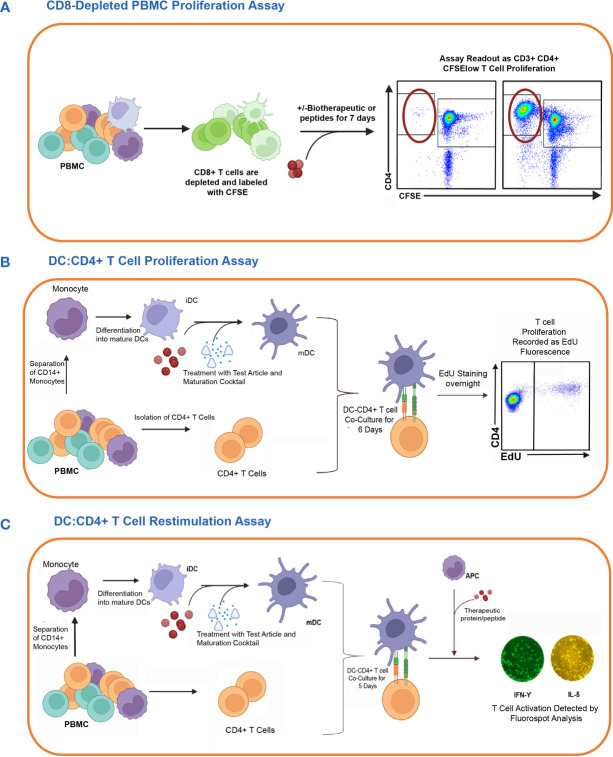
*In vitro* cell-based assay methods used to detect CD4 T cell responses to therapeutic proteins in healthy donors. The figure shows the schematic representation of three different T cell assay formats used to assess the risk of raising a CD4 T cell response. **(A)** First, the CD8 T cell depleted PBMC Proliferation Assay. Briefly, CD8 T cell depleted CFSE-labeled PBMCs are incubated for 7 days with media only, KLH, or one of the eleven therapeutic antibodies. CD3+CD4+CFSElow T cell proliferation is detected by flow cytometry analysis. **(B)** Second is the DC: CD4 T cell Proliferation Assay. Monocyte-derived DCs are exposed to the therapeutic proteins or controls and then co-cultured with autologous CD4 T cells. The proliferation of T cells in response to the activated DC is measured by flow cytometry analysis of CD3+CD4+ T cells using Click-IT^®^ EdU Cell proliferation kit. **(C)** Lastly, the DC: CD4 T cell re-stimulation assay measures the recall response of previously co-cultured CD4 T cells by re-stimulating them with or without the test articles and autologous monocytes. After 24 hours, readouts for this assay are determined by the detection of IFN-γ and IL5 cytokines by FluoroSpot. Figure created with BioRender.com.

T cell assays assess the immunogenic risk of biotherapeutics by measuring CD4 T cell activation. The complexity of the T cell assays varies from a relatively simple PBMC culture to more elaborate and time-consuming co-culture assays between monocyte-derived DCs and autologous CD4 T cells. The output of these T cell assays ranges from measuring the expression of T cell activation marker, assessing CD4 T cell proliferation, or measuring cytokine secretion via multiplexed cytokine immunoassays or ELISPOT. In this study, we evaluated three different T cell assay platforms for their capacity to predict the clinical immunogenicity of therapeutic mAbs (1): CD8 T cell depleted PBMC assay (2), DC: CD4 T cell proliferation assay, and (3) DC: CD4 T cell restimulation assay. Each assay was performed in HLA-typed PBMCs from healthy donors with keyhole limpet hemocyanin (KLH) as assay control. A schematic overview of the three CD4 T cell assays is shown in **Figure 1**.

### 3.2 Immunogenicity risk assessment using CD8 T cell depleted PBMC assay

**Figure 2:**
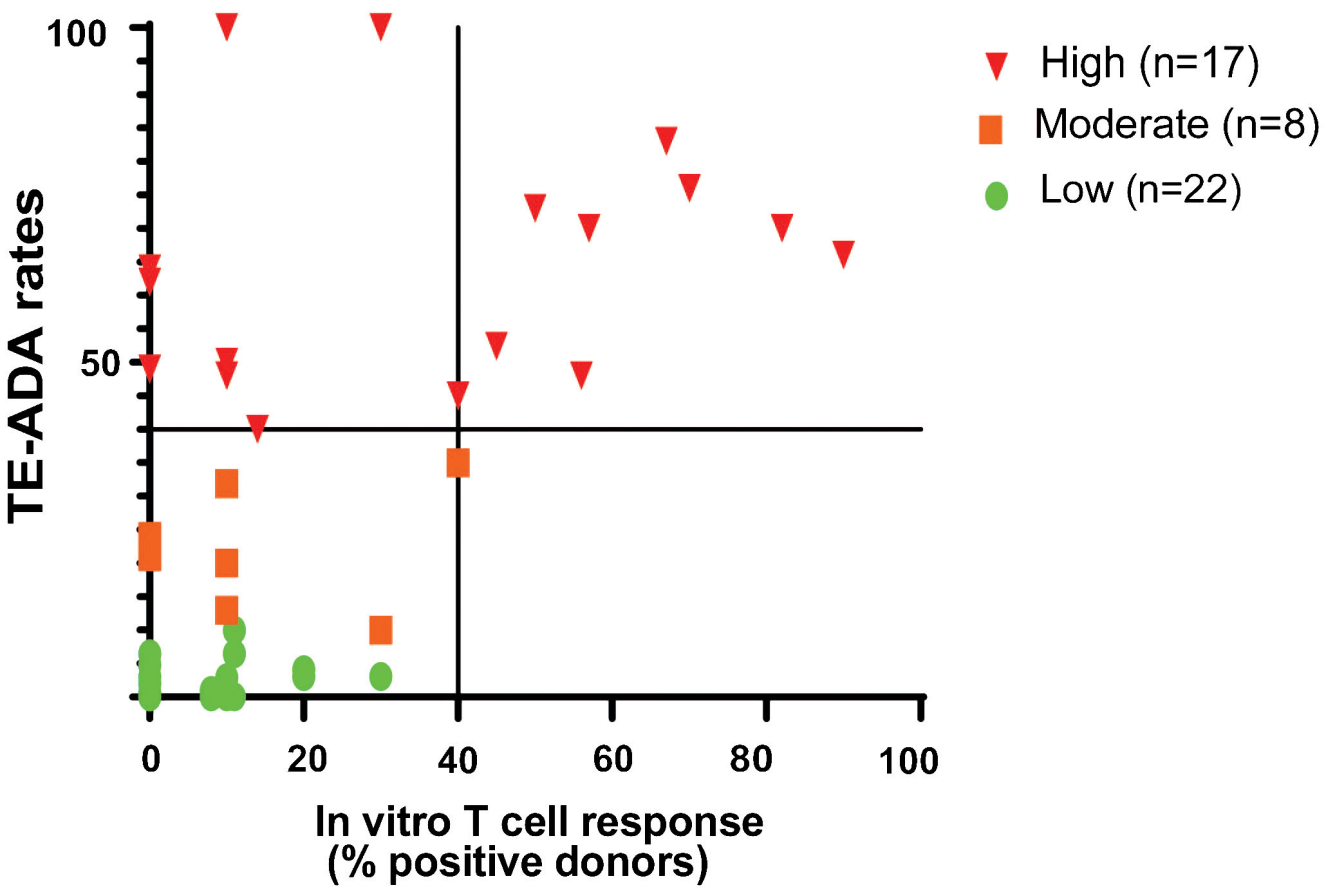
CD8 T Cell Depleted PBMC Assay Performance. The CD4 T cell Proliferation assay is suitable to detect biotherapeutics that elicit a strong CD4 T cell proliferation response (≥40% positive donor frequency) and suggests a high risk of clinical immunogenicity. The graph shows the distribution of 45 mAbs based on their performance in the CD8 T cell depleted PBMC proliferation assay compared to the clinical immunogenicity homologs with known clinical immunogenicity. mAbs were categorized into high (TE-ADA≥40%; n=15), moderate (20%<TE-ADA<40%; n=8), and low immunogenicity (TE-ADA ≤ 20%; n=23). The y-axis represents clinical immunogenicity and the x- axis depicts the evaluation of antibodies in a T cell proliferation assay.

One of the most commonly performed *in vitro* cell based assay for measuring the potential of immunogenicity is the PBMC assay (2) (**Figure 1A**). We have previously shown that a CD4 T cell proliferative assay using CD8 T cell depleted PBMC predicted the clinical immunogenicity of most of the 12 biotherapeutics tested (4). To better assess the specificity and sensitivity of this assay, we have since tested 45 homologs of mAbs and compared them with the various ADA rates in the clinic using the most up to date information available from FDA labels and publications and used the data and split the mAbs into 3 categories based on their reported clinical ADA responses in the labels or publications: high immunogenicity (treatment-emergent anti-drug antibodies (TE-ADA >40%), intermediate (20%<TE-ADA<40%) and low (TE-ADA ≤ 20%). When looking at mAbs that elicited a strong response in the CD8 T cell depleted PBMC assay (≥40% positive donors), all were classified as mAbs with either high or intermediate clinical ADAs (**Figure 2**), demonstrating the high specificity of the assay. However, only half of the mAbs with high clinical ADA (9 out of 17) elicited a response in this assay, suggesting the assay is not sensitive enough for a standalone assay for preclinical immunogenicity risk assessment (4).

**Figure 3:**
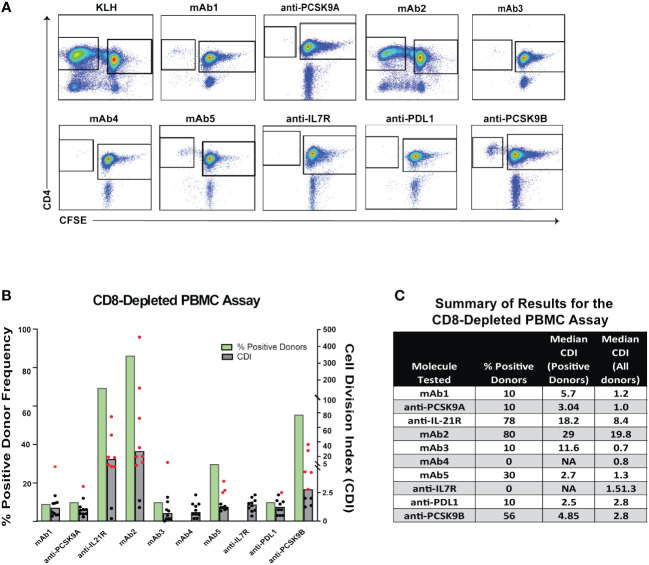
CD4 T Cell Responses to Immunogenic mAbs in CD8 T Cell Depleted PBMC Assay. **(A)** Representative plots showing flow cytometric analysis of CD4 T cell proliferative response from PBMC 7 days after incubation with media only, KLH, mAb1, anti-PCSK9A homolog, anti-IL21R homolog, mAb2, mAb3, mAb4, mAb5, anti-IL7R homolog, anti-PDL1 homolog, and anti-PCSK9B homolog. PBMCs were labeled with CFSE prior to incubation with test articles. Cells in plots were gated from DAPI^-^CD14^-^CD19^-^CD3^+^. **(B)** Bar graph summarizing the % positive donor frequency (green bars) and the magnitude of the response (grey bars represent the median CDI from 10 donors while dots represent CDIs from individual donors). If CDI ≥ 2.5, the donor is considered as positive for the tested mAb (individual red dots). Black dots represent negative donors. **(C)** Table summarizing for each mAb tested, the frequency of positive donor, median CDI of positive donors, and median CDI for all donors.

To better understand whether different T cell assay formats would have superior predictive value, we selected 10 mAbs for a comparative analysis: 2 low immunogenicity mAbs serving as negative controls, 4 high immunogenicity mAbs correctly predicted by the PBMC assay and 4 high immunogenicity mAbs that were not predicted (**Table 1**). Each mAb was evaluated in the CD8 T cell depleted PBMC assay using independent 10 donor cohorts selected based on their HLA-DR alleles to reflect the distribution of HLA types within the U.S. population. The CD4 T cell proliferative response to the mAbs was analyzed by flow cytometry after 7 days incubation using CFSE (**Figure 3A**). Individual responses were considered positive when the cell division index (CDI) was ≥2.5. With this criterion, all 10 healthy donors responded positively to the assay control KLH (Median CDI = 248.7). As expected, the low immunogenicity mAbs (mAb1 and anti-PCSK9A homolog) elicited a minimal response in the assay (1 positive donors). In contrast, the high immunogenicity mAbs ((anti-IL21R homolog, mAb2 and anti-PCSK9B homolog) elicited a CD4 T cell response in over 50% of the donors (70%, 87% and 56% positive donor frequency, respectively, **Figure 3B**). The magnitude of the T cell response in these positive donors was well above the positivity threshold of 2.5 (positive donors median CDIs of 18.2, 29, and 4.9, respectively, **Figures 3B, C**). The high immunogenicity mAb5 elicited a more moderate response in the assay with 30% positive donors and a median for positive donors of only 2.7. In contrast, four high immunogenicity mAbs (mAb3, mAb6, anti-IL7R homolog, and anti-PDL1 homolog) elicited minimal to no response in the assay. Overall, mAbs with high clinical immunogenicity exhibited a wide range of response in the CD8 T cell depleted PBMC assay.

**Table 1:** Performance of different CD4 T cell assay formats in predicting the clinical immunogenicity of therapeutic mAbs.

Biologic (mAb)	Description	Subtype	Rate of Clinical Immunogenicity	CD8 Depleted PBMC Assay %Positive Donors	DC-T Cell Assay %Positive Donors	DC-T Cell Restimulation Assay %Positive Donors
mAb1		IgG4	1%^a^	10%	0%	40%
anti-PCSK9 A	Evolocumab Homolog	IgG2	<1%^b^	10%	4%	NT
anti-IL21R	ATR-107 Homolog	IgG1	76%^a^	78%	8%	NT
anti-IL21R-IMXP	ATR-107 Homolog	IgG4	NA	NT	36%	90%
mAb2		IgG4	65%^a^	80%	54%	NT
mAb3		IgG1	90%^a^	10%	0%	10%
mAb4		IgG4	62%^a^	0%	0%	10%
mAb5		IgG1	100%^a^	30%	14%	40%
anti-IL7R	GSK2618960 Homolog	IgG1	100%^a^	0%	2%	NT
anti-PDL1	Atezolizumab Homolog	IgG1	13–54%^b^	10%	14%	NT
anti-PCSK9 B	Bococizumab Homolog	IgG2	48%^a^	56%	14%	NT

a The clinical immunogenicity rates are based on early clinical trial testing.

b The clinical immunogenicity rates are based on FDA labeling and package inserts.

Rates are based on the ADA response associated with diverse disease indications and assay testing platforms with variable sensitivity.

### 3.3 Immunogenicity risk prediction by DC: CD4 T cell proliferation assay

**Figure 4:**
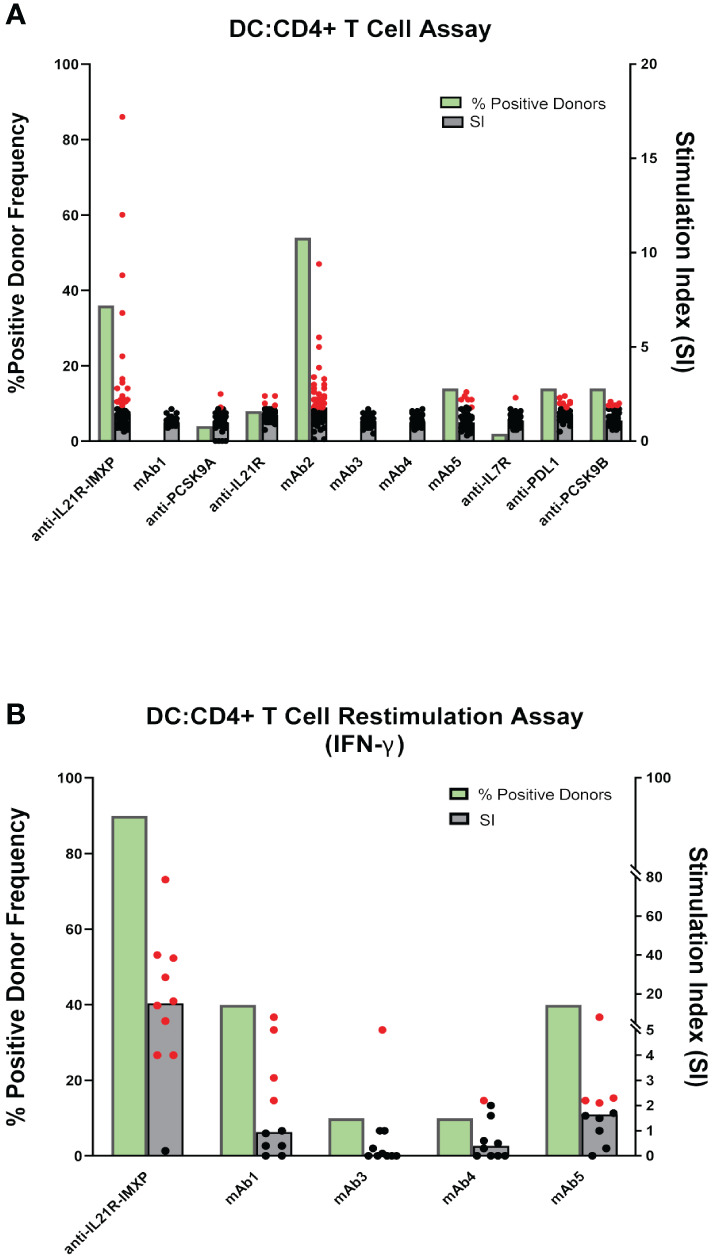
CD4 T cell responses to immunogenic mAbs in DC: CD4 T cell assays. **(A)** CD4 T cell proliferation after 6 days of co-culture of DCs pulsed with the indicated mAbs with autologous CD4 T cells. Cell proliferation was monitored by EdU incorporation. Bar graphs summarizing the percent of positive donors (green bar) and the magnitude of the response (grey bars represent the median SI from the fifty donors tested while dots represent SIs from individual donors). **(B)** IFN-γ response after 7 days of co-culture of DCs pulsed with indicated mAbs with autologous CD4 T cells and restimulation with autologous monocytes pulsed with mAbs. IFN-γ response was measured by ELISPOT. SI represents the number of IFN-γ positive cells over baseline. Bar graphs summarizing the percent of positive donors (green bars) and the magnitude of the response (grey bars represent the median SI from the ten donors tested while dots represent SIs from individual donors). If the calculated SI was above 2 (SI > 2) the donor is considered as positive for the tested mAb, represented by the red dots. Black dots represent negative donors.

One limitation of the PBMC assay is the low frequency in peripheral blood of DC, a critical cell for the initiation of CD4 T cell response. To circumvent this issue, some laboratories co-cultured monocyte-derived DC with autologous CD4 T cells to predict clinical immunogenicity. To assess the performance of the DC: CD4 T cell assay, we tested the same 10 mAbs described previously as well as a second homolog of the anti-IL21R ATR-107 (IL21R-IMXP) in 50 HLA-typed healthy donors (**Figure 1B**) selected to best represent the number and frequency of HLA-DR allotypes expressed in the US and world population (**Supplementary S1**). In this assay, the assay control KLH led to a positive response in all the donors tested (Median SI = 14) while the two negative control mAbs, mAb1 and anti-PCSK9A homolog, did not elicit a response in most donors [0% and 4% positive donors, respectively (**Figure 4A**)]. Surprisingly, the two anti-IL21R ATR-107 homologs triggered very different CD4 T cell responses. The Lilly anti-IL21R homolog elicited a weak response (8% positive donors) while the anti-IL21R-IMXP homolog triggered a response in 36% of the donors. Consistent with the PBMC assay, mAb2 induced the highest proliferative response in the DC: CD4 T cell assay (54% positive donors) but the magnitude of the response in positive donors was noticeably lower than the response triggered in the PBMC assay (median SI = 1.8). Three highly immunogenic mAbs (mAb5, anti-PDL1 homolog and anti-PCSK9-B homolog) elicited moderate responses in this assay with 14% positive donors and median SIs for positive donors hovering over 2 (2.2, 2.0, and 1.9, respectively). However, similar to what was observed for the PBMC assay, three mAbs with high clinical immunogenicity (mAb3, mAb4, and anti-IL7R homolog) triggered minimal to no response in the DC: CD4 T cell assay (0%, 0%, and 4% positive donors, respectively). Overall, the DC: CD4 T cell assay did not significantly improve the immunogenicity risk prediction for the 10 selected mAbs. With the notable exception of the anti-PDL1 homolog, mAbs that triggered a response in the DC: CD4 T cell assay also triggered a response in the PBMC assay, but the responses were of lower magnitude.

### 3.4 Immunogenicity risk prediction by DC: CD4 restimulation assay

To improve the low signal window of the DC: CD4 T cell assay, a second round of stimulation using antigen-pulsed monocytes can be added **Figure 1C** (15). To determine whether this restimulation step could improve the overall predictive value of the DC: CD4 T cell assay, we tested 5 out of the 10 mAbs: one negative control mAb1, two highly immunogenic mAbs predicted in the PBMC and DC: CD4 T cell assays ((anti-IL21R homolog, mAb5), and 2 highly immunogenic mAbs not predicted by any platform (mAb3 and mAb4). Ten healthy donors were selected based on their HLA-DRB1 alleles to reflect the U.S. population. Monocyte-derived DCs were loaded with each mAbs, matured with a cytokine cocktail, and culture with autologous CD4 T cells. After five days of co-culture, CD4 T cells were harvested and seeded onto a FluoroSpot plate with freshly isolated autologous monocytes in the presence of the respective mAb. IFN-γ Fluorospot was developed after overnight incubation. As expected, the assay control KLH triggered an IFN-γ response in all donors tested. The anti-IL21R-IMXP homolog was again the most potent mAb tested in this platform inducing an IFN- γ response in 9 out of the 10 donors tested with a median SI of 15.2 (**Figure 4B**). Surprisingly, the low immunogenicity control mAb1 and the highly immunogenic mAb5 triggered similar strong IFN-γ response (40% positive donors) while the other two immunogenic mAbs, mAb3 and mAb4 did not elicit significant response (1/10 positive donor). Overall, the restimulation step with autologous monocytes did not enhance the predictive value of the DC: CD4 T cell assay with this limited set of mAbs.

## 4 Discussion

The development of ADA of the IgG class following the administration of a biotherapeutic generally indicates that the therapeutic is driving a T-dependent immune response (16). In contrast, T-cell independent humoral immune responses that are dominated by IgMs are typically triggered by repeating polymers such as polysaccharides, glycolipids, and nucleic acids. For this reason, preclinical assays to predict clinical immunogenicity of biotherapeutics frequently rely on CD4 T cell assays. However, a wide diversity of CD4 T cell assay platforms exists with little indication of their relative performance. In this study, we compared the performance of three different CD4 T cell assays in predicting the clinical immunogenicity of 10 mAbs. This panel contained 2 non-immunogenic and 8 immunogenic mAbs. Out of the 8 immunogenic mAbs, 4 (anti-IL21R homolog, mAb2, anti-PCSK9B homolog, and mAb5) were correctly predicted by both the CD8 T cell depleted PBMC assay and the DC: CD4 T cell assay, but the magnitude of the response elicited in the DC: CD4 T cell assay was lower than the one observed in the PBMC assay. One immunogenic mAb (anti-PDL1 homolog) was only predicted by the DC: CD4 T cell assay while four mAbs (mAb3, mAb4, mAb6, and the anti-IL7R homolog) were not predicted by any of the platforms tested. Furthermore, adding a restimulation step to the DC: CD4 T cell assay did not improve the predictive value of the DC: CD4 T cell platform and instead enhanced the response to one of the negative control non-immunogenic mAb (mAb1).

One possible interpretation of the inability of the CD8 T cell depleted PBMC assay to predict the immunogenicity of some biotherapeutics could stem from the fact that DCs, key antigen-presenting cells for the initiation of the CD4 T cell response, are rare in PBMC and their numbers vary from donor to donor (15, 17). However, the lack of significant improvement in the prediction by the DC: CD4 T cell assay where antigen presentation is driven by human DC matured with inflammatory cytokines suggest that the nature of the antigen-presenting cells may not be the key issue in these assays. The only antibody that triggered a better response in the DC assay is a homolog of atezolizumab that targets PD-L1, a target that is expressed on DCs and may facilitate the antibody uptake and presentation.

Another explanation for the lack of T cell response against some immunogenic mAbs is the short duration of the assay (7 days) which may not be sufficient to efficiently stimulate the expansion of the rare antigen-specific T cells. Recent studies have shown that adding a monocyte restimulation step after the 7 days culture with DC could increase the likelihood of capturing a T cell response (15, 18). However, in our study, the restimulation step did not significantly increase the response to immunogenic mAbs and in fact enhanced the response against one of our negative controls, mAb1, that did not trigger ADA response in clinic. An alternative method that could help with the expansion of the small pre-existing CD4 T cell repertoire reactive to the drug is to add a T cell growth factor such as IL-2 during *in vitro* culture to enhance the expansion of antigen-specific CD4 T cells. Liao et al. reported a strong CD4 T cell response to the anti-IL7R GSK2618960 homolog in their PBMC assay but the assay required a 10 day-stimulation period and the presence of IL2 (19). The use of restimulation steps and T cell growth factors have been indeed very successful at promoting the expansion of drug-specific T cells (20, 21). Whether adding cytokines that promote T cell expansion in preclinical assays used for immunogenicity risk assessment will improve or hurt the predictive value of these assays is however unclear.

One of the challenges for the development and comparison of preclinical *in vitro* immunogenicity risk assays is the lack of availability of standard positive and negative control therapeutic proteins for use in assay qualification and as benchmarks for comparison of relative immunogenicity (2, 3). The different CD4 T cell responses elicited by the two anti-IL21R ATR107 homologs used in this study are an illustration of the challenge. The basis for this discrepancy is not clear and may be caused by differences in the encoding amino acid sequences, the isotype used to produce the mAb homologs, or the level of aggregates or impurities present in the two homologs. To address this issue, the Therapeutic Product Immunogenicity Community within the American Association of Pharmaceutical Scientists (AAPS) in collaboration with the Immuno-Safety Technical Committee within the Health and Environmental Sciences Institute are currently promoting the development of a reference panel of lyophilized mAbs composed of high, moderate, low immunogenicity mAbs that would facilitate cross-organization assay comparison and assay harmonization (3).

Overall, our study highlights the limitation of a preclinical immunogenicity risk assessment solely based on CD4 T cell assays with intact biotherapeutics and emphasizes the importance of additional assays to refine the preclinical immunogenicity risk assessment. The MAPPS assay that identifies MHC II-restricted peptides that are naturally presented by DCs can for example be leveraged to map potential T cell epitopes in biotherapeutics that could he be assessed for their capacity to induce CD4 T cell responses (4).

## Data availability statement

The original contributions presented in the study are included in the article/**Supplementary Material**. Further inquiries can be directed to the corresponding author.

## Ethics statement

Ethical approval was not required for the studies on humans in accordance with the local legislation and institutional requirements because only commercially available established cell lines were used.

## Author contributions

RW: Writing – original draft, Methodology, Investigation, Formal analysis, Conceptualization. AN: Writing – review & editing, Methodology, Investigation, Formal analysis. CA: Writing – review & editing, Methodology, Investigation, Formal analysis. AM: Writing – review & editing, Methodology, Investigation, Formal analysis. JS: Writing – review & editing, Methodology, Investigation. SP: Writing – review & editing, Project administration, Methodology, Investigation. LM: Writing – review & editing, Supervision, Resources, Project administration, Conceptualization.
