# Supplementary material for: Preclinical immunogenicity risk assessment of biotherapeutics using CD4 T cell assays
Source: Front Immunol. 2024 May 28;15:1406040. doi: 10.3389/fimmu.2024.1406040 (PMC11165089; doi:10.3389/fimmu.2024.1406040)
Supplement: Supplementary file 1 [file DataSheet_1.docx]

Supplementary Material

**Preclinical Immunogenicity Risk Assessment of Biotherapeutics using CD4 T cell Assays**

**Robin E. Walsh^1^, Angela Nix^1^, Chloé Ackaert^2^, Aurelie Mazy^2^, Jana Schockaert^2^, Sofie Pattyn^2^, and Laurent Malherbe^1*^**

^1^Lilly Research Laboratories, Eli Lilly and Company, Indianapolis, IN, United States

^2^ImmunXperts SA| Rue August Piccard 48, 6041 Gosselies, Belgium

* **Correspondence**:

Laurent P. Malherbe

[malherbe_laurent@lilly.com](mailto:malherbe_laurent@lilly.com)

# Supplementary Data


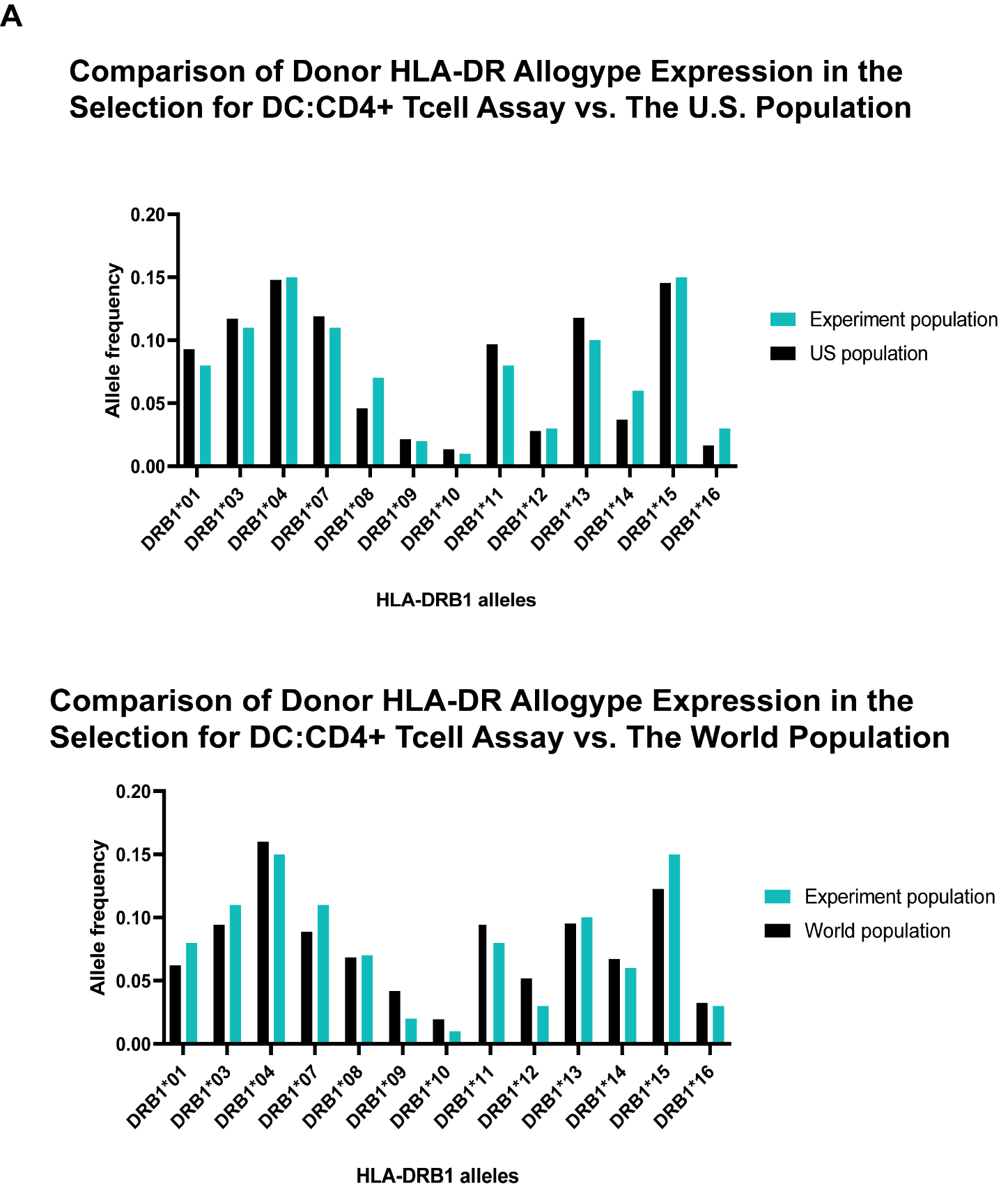


**B.**


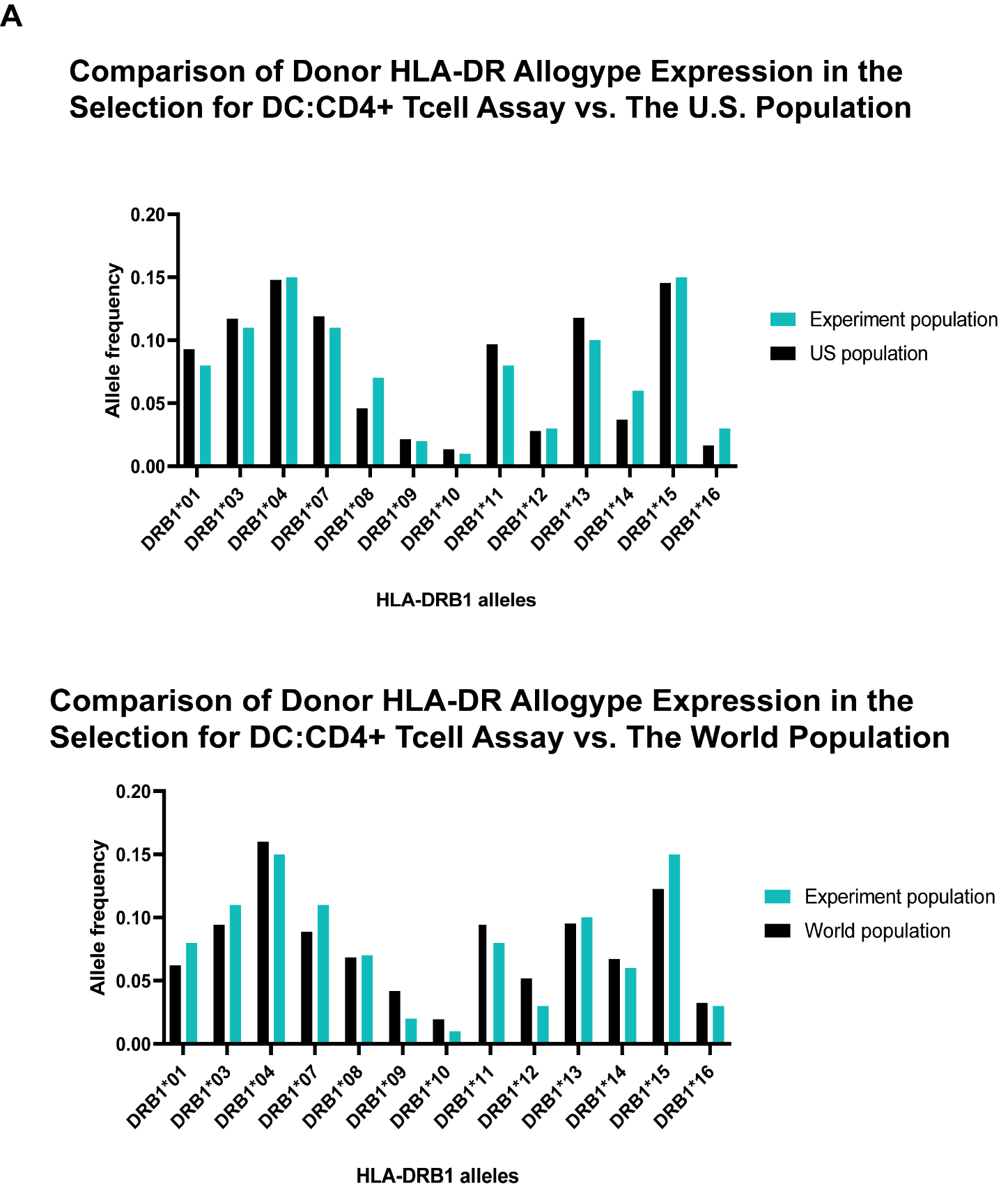


**Supplemental Figure 1.** Bar Graphs showing the relative distribution of the HLA-DRB1 type frequency of the DC:CD4+ T cell assay donor cohort. **(A)** Represents the donor cohort relative distribution to the U.S. population. (B) Represents the donor cohort relative distribution to the world population.
